# Sterol Lipid Metabolism in Down Syndrome Revisited: Down Syndrome Is Associated with a Selective Reduction in Serum Brassicasterol Levels

**DOI:** 10.1155/2012/179318

**Published:** 2012-05-09

**Authors:** Gavin Tansley, Daniel T. Holmes, Dieter Lütjohann, Elizabeth Head, Cheryl L. Wellington

**Affiliations:** ^1^Department of Pathology and Laboratory Medicine, University of British Columbia, Vancouver, BC, Canada V5Z 4H4; ^2^Institute of Clinical Chemistry and Clinical Pharmacology, University of Bonn, D-53127 Bonn, Germany; ^3^Department of Molecular and Biomedical Pharmacology, Sanders-Brown Center on Aging, University of Kentucky, Lexington, KY 40356, USA

## Abstract

Over the past 15 years, insights into sterol metabolism have improved our understanding of the relationship between lipids and common conditions such as atherosclerosis and Alzheimer's Disease (AD). A better understanding of sterol lipid metabolism in individuals with Down Syndrome (DS) may help elucidate how this population's unique metabolic characteristics influence their risks for atherosclerosis and AD. To revisit the question of whether sterol lipid parameters may be altered in DS subjects, we performed a pilot study to assess traditional serum sterol lipids and lipoproteins, as well as markers of sterol biosynthesis, metabolites, and plant sterols in 20 subjects with DS compared to age-matched controls. Here we report that the levels of nearly all lipids and lipoproteins examined are similar to control subjects, suggesting that trisomy 21 does not lead to pronounced general alterations in sterol lipid metabolism. However, the levels of serum brassicasterol were markedly reduced in DS subjects.

## 1. Introduction

### 1.1. Down Syndrome

Down Syndrome (trisomy 21) is the most common chromosomal abnormality, occurring in approximately 1 in 800 live births. DS is characterized by typical dysmorphic features, congenital abnormalities, and other medical conditions. Over the past 15 years, the life expectancy of individuals with DS has increased significantly, with the median age of death currently approaching 50 years [1], an age where the incidence of many common diseases of aging is high. Importantly, there are several differences in the way individuals with DS appear to age compared to the general population. Chief among these is the inevitable appearance of Alzheimer's Disease (AD) neuropathology by the age of 35 years [2]. Individuals with DS have also been reported to be relatively resistant to developing atherosclerosis despite the presence of an unfavorable plasma lipid profile [3]. AD and atherosclerosis are each complex, multifactorial diseases with both genetic and environmental contributors [4, 5]. As lipid metabolism contributes to the pathogenesis of both disorders [4, 5], studying lipid metabolic markers in the unique clinical situation of DS may allow our understanding of the pathogenesis and risk factors of these diseases to be refined for both the DS and the general populations.

### 1.2. Atherosclerosis in DS

Since Murdoch described a complete lack of atheroma in five institutionalized people with DS, there has been considerable interest in DS as an “atheroma-free” model [6]. Two subsequent post-mortem studies also demonstrated lower atheroma burden in institutionalized individuals with DS compared to age-matched controls [7, 8]. A recent study demonstrated reduced intima-media thickness in the carotid arteries of community-dwelling individuals with DS [9], which helped to address criticisms over the institutionalized populations used in the previous reports. These findings are particularly striking in light of the fact that individuals with mental retardation are typically at increased risk for atherosclerosis [10]. Indeed, the hypothesis that individuals with DS are protected from the development of atherosclerosis is interesting, but an explanation for this observation has not been elucidated to date.

Atherosclerosis is a complex, progressive inflammatory disorder in which dysregulated lipid metabolism plays a central role [5]. The causal link between circulating cholesterol levels and atherosclerosis is well established. For example, elevated levels of low-density lipoprotein cholesterol (LDL-C) definitively increase atherosclerosis risk [11, 12]. LDL, which transports cholesterol from the liver to peripheral tissues, satisfies all of Koch's modified postulates and has a causal role in the pathogenesis of atherosclerosis [13]. This role is best illustrated by the success of statins and other cholesterol lowering medications in reducing LDL-C levels, thereby decreasing the number of cardiovascular events in treated patients [14]. Not surprisingly, however, given the complexity of atherosclerotic disease, lipoproteins other than LDL may also contribute.

High-density lipoprotein (HDL) is the plasma lipoprotein that mediates reverse cholesterol transport, a process that extracts excess cholesterol from peripheral tissues and transports it to the liver to be ultimately excreted as bile [15]. Elevated levels of HDL-C have been clearly shown to be protective against the development of atherosclerosis even in the context of high LDL-C levels [11, 16]. Through intense investigations on HDL biogenesis and function, several members of the ATP binding cassette (ABC) superfamily have been characterized. ABCA1 and ABCG1 are genes that encode for proteins involved in the efflux of cholesterol from peripheral cells onto HDL [17]. ABCA1 catalyses the initial transfer of lipids onto apolipoprotein A-I (apoA-I), which is the rate-limiting step in the formation of nascent HDL particles [18]. ABCG1 continues this process of adding lipids to HDL [18]. Notably, ABCG1 localizes to the long arm of chromosome 21 [19] and is inherited in triplicate in most people with DS, raising interesting questions about whether excess ABCG1 may underlie some of the differences in lipid metabolism in this group compared to the typically developing population. Intriguing new data from preclinical studies show that ABCG1 also has important roles in endothelial function, where it promotes oxysterol efflux and protects from hypercholesterolemia-mediated endothelial dysfunction [20]. Conversely, genetic deficiency of ABCG1 in mice promotes endothelial activation, enhances monocyte adhesion and increases vascular inflammation [21]. Although these mechanisms have yet to be examined in DS subjects, abundant ABCG1 function in the endothelium may help to explain their relative protection from atherosclerosis.

Studies of plant sterols have further expanded our knowledge of the role of lipids in the progression of atherosclerosis. Because a high intake of plant sterols reduces circulating cholesterol levels [22], functional foods enriched in plant sterols are now offered commercially as a means to lower total plasma cholesterol levels. Plant sterols differ from cholesterol by the presence of a double bond at C22 and/or a methyl or ethyl group at position C24 [22]. The major plants sterols include campesterol (methyl C24), sitosterol, (ethyl C24), brassicasterol (D22, methyl C24), and stigmasterol (D22, ethyl 24). In the enterocyte, plant sterols are believed to compete with cholesterol for incorporation into micelles, thereby reducing cholesterol absorption [22]. Several lines of evidence suggest that increased levels of circulating plant sterols correspond with a decreased risk for cardiovascular disease. Ntanios et al. have shown hamsters fed a diet enriched in phytosterol esters have significantly fewer foam cells, suggesting that phytosterols inhibit a key step in the progression of atherosclerosis [23]. Fassbender et al. have also shown an association between elevated circulating plant sterols and a reduced tendency towards symptomatic atherosclerosis in a Dutch cohort [24].

However, other observations complicate this issue, the most notable of which is that sitosterolemic patients, who have markedly increased circulating sitosterol levels, exhibit accelerated atherogenesis [25]. Sitosterolemia is a rare autosomal recessive condition caused by mutations in two other ABC transporters: ABCG5, and ABCG8 [26]. Both ABCG5 and ABCG8 are expressed exclusively in the enterocyte and efflux plant sterols from the enterocyte back into the intestinal lumen, thereby reducing plant sterol absorption [27]. In sitosterolemia, lack of ABCG5/ABCG8 function leads to significantly greater absorption of dietary cholesterol and sitosterol and an increased incidence of cardiovascular events independent of plasma LDL-C levels, suggesting that sitosterol itself may be contributing to atheroma formation [25]. A recent *in vitro* study also showed differential effects of various plant sterols, both protective and deleterious, on ABC transporter expression in foam cells [28]. There is currently no clear consensus on the contributions of plant sterols to the development of atherosclerosis, but recent research suggests that numerous pathways may be involved. It is not yet known whether plasma levels of plant sterols in people with DS differ from the general population.

In attempts to better understand the pathogenesis of atherosclerosis in individuals with DS, several groups have investigated the traditional atherosclerotic risk factors of circulating total cholesterol (TC), LDL-C, and HDL-C in the DS population. Although these studies all vary significantly in their sample sizes, specific outcomes, and control groups, they nonetheless provide some useful insights into the traditional atherosclerotic risk profiles of the DS population. One study demonstrated a favorable lipid profile in individuals with DS, yet noted elevated levels of homocysteine, which has been suggested to increase atherosclerosis risk [29]. Several studies found no change in serum LDL-C or HDL-C in individuals with DS compared to a control group or to population norms [9, 30–32]. Many other studies demonstrated an increased number of atherosclerotic risk factors in individuals with DS. For example, Draheim et al. observed elevated triglycerides and total body fat in their DS study group [31]. These findings agree with a much earlier study by Nishida et al. [32] and are consistent with our current understanding of the metabolic syndrome, which is common in DS. Other studies have also observed a lipid profile that would suggest individuals with DS to be at an increased risk for atherosclerosis [29, 33]. These widely variable results underscore the lack of consensus as to whether lipid profiles of individuals with DS vary from the general population and whether they confer increased atherosclerotic risk. Notably, factors known to correlate with atherosclerosis in the general population, such as fruit and vegetable intake, serum LDL-C, and smoking status, all correlate poorly with intima-media thickness in individuals with DS [9]. This surprising result, in addition to the conflicting data surrounding traditional atherosclerotic risk factors, suggests that there may be some distinct mechanisms underlying atheroma formation in DS.

### 1.3. Alzheimer's Disease in DS

AD is the most common form of dementia in the elderly and currently affects over 50% of the general population greater than 85 years of age [4]. The vast majority (96%) of AD patients begin to experience memory dysfunction in their 60s–80s [4]. The remaining patients carry genetic mutations that lead to an early-onset familial form of AD, which can manifest as early as the mid 30s [34]. All AD patients develop two neuropathological hallmarks including amyloid plaques that consist of aggregated A*β* peptides and neurofibrillary tangles that contain hyperphosphorylated tau protein [35]. As detailed below, the 2011 guidelines for the diagnosis of AD recognize that changes in A*β* and tau metabolism begin to occur decades earlier than the onset of cognitive dysfunction [36].

It is well established that DS subjects inevitably develop amyloid and tau deposits by their mid 30s [37]. As amyloid precursor protein (APP), the protein from which A*β* peptides are derived, is on chromosome 21, inheritance of excess APP has been thought to underlie the accelerated onset of AD in the DS population although this is not universally accepted [38–41]. However, additional other mechanisms, many of which are related to lipid metabolism, may also contribute.

First, the levels and distribution of intracellular cholesterol can affect several aspects of APP and A*β* metabolism. For example, the proteolytic processing of APP into A*β* is highly influenced by intracellular cholesterol levels such that excess cholesterol increases A*β* production whereas cholesterol depletion minimizes it [42–45]. Once produced, A*β* peptides are degraded, often within microglia, and recent studies show that excess intracellular cholesterol in microglia delays A*β* degradation whereas cholesterol efflux from microglia promotes A*β* degradation [46].

Second, apolipoprotein E (apoE) is one of the protein components of chylomicrons and very low-density lipoproteins in plasma, as well as the major apolipoprotein in the brain [47]. The human APOE gene encodes three alleles, apoE2, apoE3, and apoE4. In 1993, apoE genotype was identified as a genetic risk factor for late onset AD [48, 49] and to this day remains the most robust genetic risk factor for late onset AD in the general population [50]. Although exactly how apoE4 contributes to increased AD risk is not entirely understood, apoE is known to bind A*β* and facilitates its proteolytic degradation, with apoE4 as less efficient in A*β* clearance than either apoE2 or apoE3 [46]. Increasing the lipid content on apoE facilitates A*β* clearance both *in vitro* and *in vivo* [51–55].

Third, epidemiological evidence suggests that plasma lipid levels are associated with AD risk. Specifically, high LDL-C and/or low HDL-C levels, particularly in midlife, have repeatedly been associated with AD risk [56–60]. Understanding the association between circulating cholesterol levels and AD risk has been challenging because neither cholesterol nor apoE crosses the blood brain barrier (BBB) [61]. However, apoA-I, the major apolipoprotein on HDL, is capable of BBB transit and has recently been shown to markedly affect cerebrovascular amyloid levels and cognitive function in mouse models of AD [62, 63]. Recently, lipidomic approaches suggest that decreased plasma sphingomyelin and increased plasma ceramide mass correlate with cognitive function in AD [64].

Finally, retrospective epidemiological studies suggest that statins, drugs that inhibit the rate-limiting step in cholesterol biosynthesis and are widely used to lower LDL-C levels, reduce AD prevalence [65–67]. Although subsequent prospective, randomized-controlled trials of statins failed to show efficacy in the ability of statins to either prevent or treat AD [68, 69], these trials, like many in the AD field, may have failed because treatment was initiated past the therapeutic window.

Identifying molecular and biochemical changes of AD early in the disease process is necessary to allow treatment to begin prior to neuronal loss and cognitive decline. To better define this therapeutic window, in 2011 the National Institutes of Health (NIH) released new clinical guidelines for the diagnosis of AD that incorporate the current understanding of early stages in AD pathogenesis [36]. The first detectable changes associated with AD are alterations in specific proteins in the cerebrospinal fluid (CSF), specifically decreased levels of A*β* 1–42 and increased levels of phosphorylated tau protein. This is followed by development of amyloid deposits in the brain, which can be visualized in a living patient using positron emission topography (PET) with a specific amyloid ligand known as Pittsburgh compound B (PIB). Neuronal atrophy, which follows a distinct pattern, later becomes detectable by magnetic resonance imaging (MRI). Finally, cognitive problems emerge. The sequence of AD pathology can begin up to 20 years prior to the onset of cognitive symptoms [70]. Clearly, these new guidelines represent an enormous advance on our ability to track the onset and progression of AD in the general population.

Very recently, Vanmierlo et al. demonstrated that the levels of the plant sterol brassicasterol were significantly reduced in the CSF of cognitively impaired AD subjects with an intact BBB [71]. Because plasma brassicasterol levels were unchanged, reduced CSF brassicasterol levels were hypothesized to reflect altered choroid plexus function during the progression of AD [71]. Importantly, in this study, CSF brassicasterol levels improved the predictive power of the other validated CSF biomarkers, A*β* and tau. Although their study was not designed to determine whether CSF brassicasterol levels may be prognostic of AD progression, their observations nonetheless generate interesting hypotheses about the utility of plant sterol metabolism as a potential biomarker in AD.

## 2. Study Design

### 2.1. Subjects

Twenty community-dwelling subjects with DS were assessed in this pilot study (Table 1). DS subjects were identified from the University of Irvine California clinic or group homes, all had clinical features of trisomy 21 and nearly all had karyotypic analyses confirming trisomy 21. The 22 typically developing control subjects did not have trisomy 21 and were excluded for AD, diabetes and obesity. Clinical Research Ethics Boards from the University of Irvine, California and the University of British Columbia approved this pilot study. Written informed consent was obtained from each DS subject or caregiver and control subject.

### 2.2. Plasma Lipid, Lipoprotein, and CRP Analysis

TC and HDL-C were measured from nonfasting serum by enzymatic kits (Wako) according to the manufacturer's protocols. ApoA-I and apolipoprotein B100 (apoB) were measured using an immune-nephelometric assay on the Siemens ProSpec automated analyzer (Siemens Diagnostics, Tarrytown, NY). The maximum interassay coefficient of variation (CV) of the assay is 2.2% and 1.9% for apoA-I and apoB, respectively. CRP was measured with a enzymatic chemiluminescent immunometric assay using the Siemens IMMULITE 2500 automated analyzer. The linear range of the assay is 0.2–150 mg/L, with a maximum interassay CV of 8.7%.

### 2.3. Sterol Extraction and Analysis from Plasma

Samples were frozen in aliquots and stored at −80°C until analysis. Serum concentrations of cholesterol were measured by gas chromatography-flame ionization detection using 5*α*-cholestane as internal standard. The cholesterol precursors lanosterol, dihydrolanosterol, lathosterol, and desmosterol, the plant sterols campesterol, brassicasterol, sitosterol, and stigmasterol as well as the 5*α*-saturated compounds cholestanol, campestanol and sitostanol were measured by a modified sensitive method using combined gas chromatography-mass spectrometry (GC-MS) using epicoprostanol as internal standard. The cholesterol oxidation products, 7*α*-, 24S-, and 27-hydroxycholesterol, were measured by GC-MS isotope dilution methodology using deuterium, that is, stable isotope labeled 7*α*-, 24R, S-, and 27-hydroxycholesterol as internal standards [72].

Fifty *μ*g 5*α*-cholestane (Serva) (50 *μ*L from a stock solution of 5*α*-cholestane in cyclohexane; 1 mg/mL) and 1 *μ*g epicoprostanol (Sigma) (10 *μ*L from a stock solution epicoprostanol in cyclohexane; 100 *μ*g/mL) were added to 100 *μ*L serum. One mL NaOH (1 M) in 80% ethanol was added and the alkaline hydrolysis was performed for 60 min at 61°C. The sterols were subsequently extracted with 3 mL of cyclohexane twice. The organic solvents were evaporated and the residual plasma sterols were dissolved in 160 *μ*L n-decane. Eighty *μ*L of the serum n-decane samples were transferred into microvials for gas-liquid chromatography-mass spectrometry—selected ion monitoring (GC-MS) of sterols, stanols and oxysterols. The sterols and stanols were derivatized to trimethylsilyl (TMSi) ethers by adding 10 *μ*L TMSi-reagent (pyridine : hexamethyldisilazane-trimethylchlorosilane; 9 : 3 : 1, by volume; all reagents were applied from Merck) and incubated for 1 h at 64°C.

The residual 80 *μ*L of the serum n-decane samples were diluted with 300 *μ*L n-decane and derivatized with 30 *μ*L TMSi-reagent preceding analysis of cholesterol by gas chromatography-flame ionization detection (GC-FID).

### 2.4. GC-FID and GC-MS

Plasma cholesterol was quantified by GC-FID on an HP 6890 series II plus GC (Agilent Technologies, Böblingen, Germany) using 5*α*-cholestane as an internal standard. An aliquot of 2 *μ*L was injected in a splitless mode at 280°C by an automated sampler and injector (HP 7683). Hydrogen was used as carrier gas with an inlet pressure of 9.9 psi, resulting in a total gas-flow of 1.1 mL/min and the temperature of the flame ionization detector was kept at 280°C. The sterols were separated on a cross-linked methyl silicone DB-XLB 122-1232 fused silica capillary column (J&W, Folsom, USA) (30 m × 0.25 mm i.e., × 0.25 *μ*m film thickness) in an Hewlett-Packard (HP 6890) gas chromatograph. The oven temperature was initially kept at 150°C for 3 min, and then gradually increased to a final temperature of 290°C. The ratios of the cholesterol areas to the area of internal standard were calculated and multiplied by the added amount of the internal standard (50 *μ*g 5*α*-cholestane) to reveal absolute cholesterol concentrations.

GC-MS was performed on an HP GC-MSD system (HP 5890 series II GC) combined with a 5971 mass selective detector (Agilent Technologies, Böblingen, Germany) equipped with a DB-XLB 122-1232 fused silica capillary column (J&W, Folsom, USA) (30 m × 0.25 mm i.e., × 0.25 *μ*m film thickness) in the splitless mode using helium (1 mL/min) as the carrier gas. The temperature program was as follows: 150°C for 1 min, followed by 20°C/min up to 260°C, and 10°C/min up to 280°C (for 15 min). The sterols, stanols, and oxysterols were monitored as their TMSi derivatives in the selected ion monitoring mode using their characteristic masses [73]. Identity of all sterols was proven by comparison with the full-scan mass spectra of authentic compounds (range, m/z 50–500). All the above sterols, stanols, and oxysterols were sufficiently separated on the column from each other. Accuracy of the method was established by recovery experiments, day to day variation (below 3%), limit of detection and limit of quantification below the present concentrations for each sterol.

### 2.5. Statistics

Data were analysed by unpaired two-tailed Student's *t*-test (GraphPad Prism v5.0), applying Welch's correction when variances were significantly different between groups. A *P* < 0.05 was considered statistically significant.

## 3. Results

### 3.1. Study Subjects

A total of 20 community-dwelling DS and 22 healthy, typically developing control subjects were recruited for this pilot study. The complete cohort (Table 1) did not differ statistically in mean age (*P* = 0.167) despite a wider age range in the control compared to the DS cohort. Because TC and HDL-C levels vary by age, we also divided each cohort into two groups aged <45 years and >45 years (Table 2) with no significant differences in mean age (*P* = 0.088 for <45 years, *P* = 0.131 for >45 years). However, there are several caveats associated with our cohorts for this pilot study. First, the DS cohort has significantly more males than the control group, in both the pooled sample and after dividing each cohort into two age groups. Second, control and DS subjects were not matched for body mass index, diabetes, diet, and exercise, all of which pose significant potential confounds to our pilot results. Nevertheless, this pilot study yielded some interesting observations that could be used to design a future investigation that is sufficiently powered and adequately matched for these variables.

### 3.2. Serum Lipids, Lipoproteins, Sterol Precursors, and Metabolites in the Pooled Sample

No significant differences were observed between control and DS subjects with respect to TC, HDL-C, apoA-I, and apoB levels (Table 2). Consistent with this, the levels of cholesterol biosynthetic intermediates lathosterol, lanosterol, dihydrolanosterol, and desmosterol also showed no significant differences between DS and control groups. Cholesterol itself did not differ from controls either when measured by an enzymatic assay or by GC-MS (Table 2). Similarly, the levels of the rate-limiting bile acid biosynthetic marker 7*α*-OH-cholesterol were unchanged between DS and control groups (Table 2). Taken together, these observations suggest that global cholesterol homeostasis is not significantly altered by trisomy 21.

In humans, the cholesterol metabolite 24S-OH-cholesterol is exclusively generated in the brain by the enzyme cyp46 and plays an important role in maintaining cholesterol homeostasis in the CNS [74, 75]. 24S-OH-cholesterol easily crosses the BBB and its serum levels are therefore a marker of brain cholesterol turnover. Serum 24S-OH-cholesterol levels are not significantly different between DS and control subjects, suggesting that DS is not associated with altered cholesterol catabolism in the CNS. In contrast to 24S-OH-cholesterol, the ubiquitous oxysterol 27-OH-cholesterol is significantly reduced in DS subjects compared to controls (*P* = 0.004).

**Figure 1:**
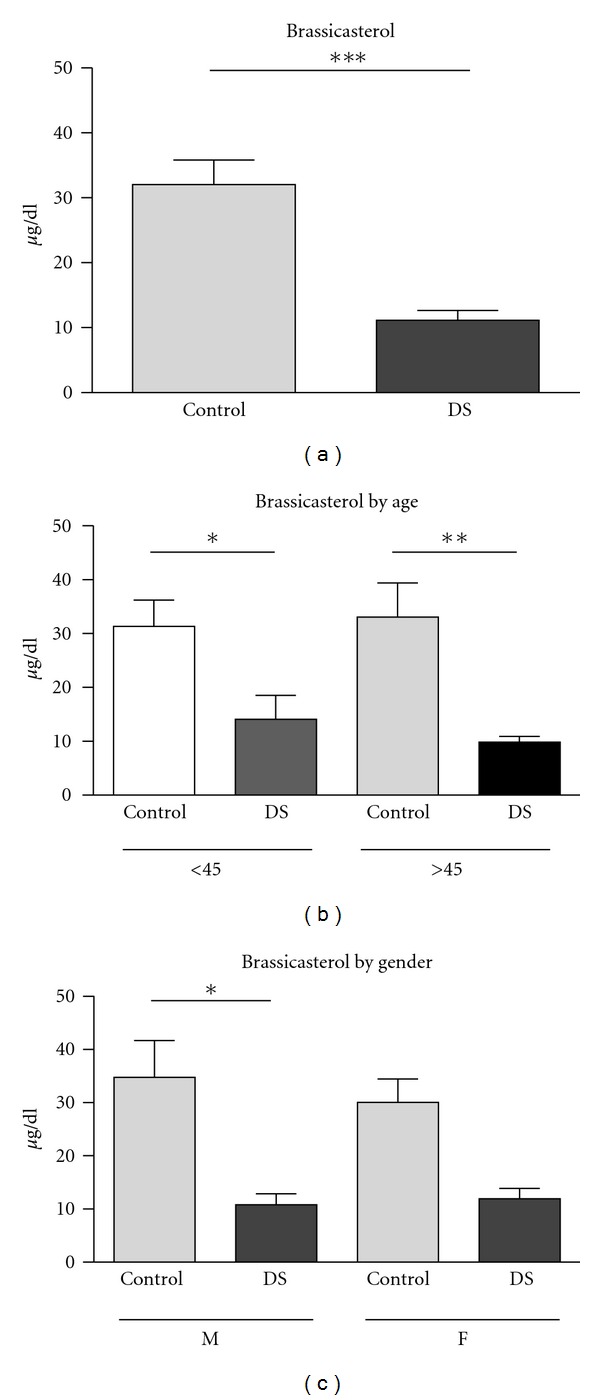
Serum brassicasterol levels are reduced in Down Syndrome (DS) subjects. Serum brassicasterol levels were quantified by GC-MS in *N* = 20 DS and *N* = 22 healthy controls (CON). (a) Mean and standard error of brassicasterol levels in the total CON and DS sample. (b) Mean and standard error of brassicasterol levels in the CON and DS subjects <45 and >45 years of age. (c) Mean and standard error of brassicasterol levels in male and female CON and DS subjects. Data were analysed by Students *t*-test. *represents *P* < 0.05, **represents *P* < 0.01, and ***represents *P* < 0.001.

The inflammatory marker CRP was significantly elevated in DS subjects compared to controls (*P* = 0.035), as was the sterol metabolite cholestanol (*P* = 0.010). Although the levels of the plant stanols campestanol and sitostanol were unchanged between DS and control subjects, we observed that serum levels of most of the plant sterols evaluated were reduced. Most strikingly, brassicasterol levels were remarkably lower in DS subjects (*P* < 0.0001) (Table 2, Figure 1(a)). Sitosterol levels are also significantly lower in DS subjects (*P* = 0.025) and campesterol levels show a nonsignificant trend toward reduced levels in DS subjects (*P* = 0.056). Stigmasterol levels were comparable in DS and control subjects (*P* = 0.092).

### 3.3. Serum Lipids, Lipoproteins, Sterol Precursors, and Metabolites by Age

When divided into two groups aged <45 and >45 years, no significant differences were observed between control and DS subjects with respect to TC, HDL-C, and apoB levels (Table 3). ApoA-I levels, which were not different in the pooled sample, were significantly reduced only in DS subjects >45 years compared to controls (*P* = 0.016) (Table 3). When measured by GC-MS, cholesterol levels were significantly elevated only in DS subjects <45 years (*P* = 0.008). Similar to the pooled sample, the levels of sterol precursors lathosterol, lanosterol, dihydrocholesterol, and desmosterol did not differ when adjusted by age between DS and control subjects, nor were 7*α*-OH-cholesterol levels changed in either age group (Table 3). Also consistent with the pooled sample, 24S-OH-cholesterol levels remained unchanged when DS and controls subjects were stratified by age (Table 3). Serum 27-OH-cholesterol levels remained significantly lower only in DS subjects <45 years (*P* = 0.010, Table 3). After dividing by age, CRP levels significantly elevated only in subjects <45 years compared to controls (*P* = 0.051). Similarly, elevated levels of cholestanol were retained only in DS subjects <45 years (*P* = 0.022).

Consistent with the pooled sample, levels of the plant stanols campestanol and sitostanol remained unchanged when DS and control subjects were grouped by age. When stratified by age, brassicasterol levels remained greatly reduced in DS subjects <45 years (*P* = 0.039) as well as in DS subjects >45 years (*P* = 0.007) compared to age-matched controls (Table 3, Figure 1(b)). Sitosterol levels also remained significantly lower in DS subjects >45 years (*P* = 0.010) and campesterol levels were also significantly reduced in this age group of DS subjects (*P* = 0.014) compared to controls. Similar to the pooled groups, stigmasterol levels were comparable in DS and control subjects at each age examined (<45 y: *P* = 0.280, >45 y: *P* = 0.572).

### 3.4. Serum Brassicasterol Levels Are Significantly Reduced in Both Male and Female DS Subjects

Of all of the analytes examined, brassicasterol appears to have the most robust association with DS, being dramatically lower in both the pooled sample and in each age group. However, because a major caveat of our study is that the DS cohort had significantly more male subjects, particularly for the group >45 years, we also analysed serum brassicasterol levels independently in male and female DS subjects compared to controls. We observed significantly lower brassicasterol levels in male (*P* = 0.009) and female (*P* = 0.002) DS subjects compared to controls (Figure 1(c)).

## 4. Discussion

Although atherosclerosis is a multifactorial disease with environmental factors integral to its progression, several lines of evidence suggest that environmental differences alone cannot explain the apparent resistance to atherosclerosis in the DS population. The pathology reports that first described DS as an atheroma-free population compared DS subjects to other institutionalized controls [6]. Presumably, factors such as exercise, diet, and other environmental variables were comparable between these two non-community-dwelling groups, suggesting that their different atheroma burden may be independent of environmental factors [6–8]. Through dietary surveys given to a group of individuals with DS residing in the community, Braunschweig et al. concluded that people with DS consumed a comparable diet to that of the general population [30].

Being a pilot study, our results have several associated caveats. Our sample is small in size, poorly matched for gender, and lacks nutritional information. Because our goal was to compare traditional sterol lipid profiles between people with DS and healthy typically developing controls, we did not specifically recruit a group of controls with normal intellectual ability who were matched for body mass index and diet with DS subjects. This will be an essential additional control group for future studies. The lipids to be analysed in the future should be expanded to include cholesteryl esters, sphingomyelin, gangliosides, and lipids involved in signaling, as these factors can contribute to an overall change in lipidomic profiles in both AD and atherosclerosis and may offer additional insights into how such lipids may affect AD or cardiovascular risk [76, 77].

Despite these caveats, our pilot investigation suggests that the traditional atherosclerotic risk factors in people with DS generally do not differ from typically developing controls and are in accordance with several previous studies [9, 30–32]. Specifically, TC, HDL-C, and apoB were comparable in all of our subsets. Although differences in apoA-I and cholesterol measured by GC-MS did reach statistical significance in some subsets, these changes were not retained across age groups or genders. These observations, in addition to the finding that markers of cholesterol biosynthesis and metabolism are also not significantly altered in DS subjects, suggest that the overall sterol metabolic profile in DS is similar to the general population. Our study revealed that cholestanol levels were increased in younger subjects with DS, whereas 27-OH levels were reduced, but the significance of these observations is not immediately obvious. Our study is the first to test for alterations in circulating 24S-OH-cholesterol levels in subjects with DS compared to controls. In humans, 24S-OH-cholesterol is exclusively generated in CNS neurons and easily crosses the BBB, where its levels in the circulation reflect sterol turnover in the CNS [75]. Again, no changes were observed, suggesting that overall sterol metabolism in the DS brain is similar to that in the general population.

The major conclusion of our pilot study is that serum brassicasterol levels are significantly reduced in people with DS. The mechanism responsible for this change is unclear, as there is no obvious physiological explanation for this finding. However, the reduction is robust and persists across genders and age groups. Because plant sterols are entirely derived from the diet, the lack of dietary information in the DS and controls subjects studied here is an obvious caveat to our study. However, we believe that dietary differences alone are unlikely to entirely account for the reduced brassicasterol levels in DS subjects, as community-dwelling individuals with DS have previously been shown to consume similar diets as control subjects [30] and that other diet-derived plant sterols studied here were not significantly altered between DS and control subjects. Our findings raise interesting questions about the mechanisms underlying reduced brassicaterol levels and the effects of low brassicaterol levels on atherosclerosis risk. Plant sterols have atheroprotective properties attributed to their ability to reduce cholesterol absorption, yet TC, HDL-C, apoB and most plant sterol levels were comparable between DS and control subjects. If anything, low brassicaterol levels would argue in favor of increased risk and raise questions about whether different plant sterols have distinct effects.

Although much remains to be explored in this area, it is likely that genetic factors in DS may have a larger effect compared to environmental factors in modulating atherosclerosis risk. Fetal tissues have been used to determine whether trisomy 21 leads to genetically determined differences in lipid metabolism between DS and control subjects, as the blood-placental barrier maintains separation between the fetal and maternal circulation [78]. Therefore, nearly all cholesterol present in fetal blood is synthesized by the fetus itself. One study showed that serum TC levels are elevated in fetuses with DS, and a follow-up study by the same authors demonstrated elevated cholesterol levels in DS fetal liver samples [79, 80]. Although more work needs to be done to better define the metabolic differences in DS, this area has considerable potential to establish genetically-defined baseline lipid and lipoprotein levels in people with DS. One particularly promising area of research may be to investigate the role of ABCG1 on endothelial function in DS, given that studies in preclinical models suggest that excess ABCG1 may promote endothelial resistance to triggers of atherosclerosis [20, 21]. Although there is no firm consensus, some preclinical studies support a role for ABCG1 in atherosclerosis in specific animal models [81–84]. However, none of these studies specifically investigated endothelial function in their model systems. It is conceivable that the possible protection from atherosclerosis in the DS population may not be strongly associated with plasma lipid levels but rather may be due to better endothelial function.

An important question raised by our pilot study is whether brassicaterol levels in the CSF or brain tissue of DS subjects is altered compared to controls. Decreased CNS brassicaterol levels in DS would support the previous association with validated CSF A*β* and tau biomarkers for AD in the general population [71]. An important consideration for further investigation in this area is to include young DS subjects, as our subject group is of a mean age that would already invariably exhibit AD neuropathology. It is unclear, in both the general and DS populations, if changes in brassicasterol levels precede the development of AD neuropathology or dementia. The new AD diagnostic guidelines offer an unprecedented ability to study how the trajectory of AD pathogenesis may differ in DS subjects. Our preliminary results suggest that inclusion of CNS brassicasterol measurements may also add to the possible understanding of AD pathogenesis in the unique DS population.

## 5. Conclusions

In people with DS, standard serum markers of sterol lipid metabolism are generally unchanged from age-matched controls and offer little insight into why DS subjects appear to have reduced prevalence of atherosclerotic disease. Further investigation into ABCG1 function, which is inherited in triplicate in trisomy 21 and plays roles in HDL metabolism and endothelial function, may prove more informative. Among the many analytes examined in our study, serum levels of the plant sterol brassicasterol levels were remarkably reduced in DS subjects relative to healthy controls across age and gender. As CSF brassicasterol levels have been reported to be reduced in AD patients and to improve the predictive power of CSF A*β* and tau levels as AD biomarkers, it will be of interest to determine whether serum and CSF brassicaterol levels are reduced in DS subjects throughout their lifespan or could be used as a prognostic biomarker of incipient AD neuropathology in DS subjects.

## Figures and Tables

**Table 1:** Demographics of control and DS cohorts.

	Con < 45	DS < 45	Con > 45	DS > 45	Con	DS
*N*	13	6	9	14	22	20
% male	61	83	22	64	45	70
Mean age (range)	38.92 (29–44)	42.53 (39–44)	50.78 (46–61)	47.8 (45–49)	43.77 (29–61)	46.29 (39–49)

**Table 2:** Serum analytes in the total control and total DS cohorts. Analytes shown in bold are significantly different between control and DS groups by unpaired Student's *t*-test. Welch's correction was applied when variances were significantly different between groups.

	Con	DS	*P*
TC (mmol/L)	4.858 (±0.743)	4.925 (±1.123)	0.834
HDL-C (mmol/L)	1.591(±0.514)	1.412 (±0.271)	0.116
ApoA-I (mg/mL)	1.4266 (±0.245)	1.324 (±0.179)	0.051
ApoB (mg/mL)	0.8028 (±0.176)	0.9015 (±0.255)	0.178
CRP (mmol/L)	**1.361 (** ± **1.767)**	**2.847 (** ± **2.101)**	**0.035**
Campesterol (mg/dL)	0.4953 (±0.279)	0.3424 (±0.194)	0.056
Sitosterol (mg/dL)	**0.4040 (** ± **0.196)**	**0.2716 (** ± **0.153)**	**0.025**
24S-OH cholesterol	64.06 (±2.540)	69.90 (±5.199)	0.296
Lathosterol (mg/dL)	0.1930 (±0.097)	0.225 (±0.130)	0.401
Campestanol (mg/dL)	3.552 (±1.385)	3.368 (±1.811)	0.729
Stigmasterol (*μ*g/dL)	9.721 (±0.602)	11.93 (±1.115)	0.092
Sitostanol (mg/dL)	4.586 (±0.255)	4.347 (±0.436)	0.639
Lanosterol (*μ*g/dL)	17.50 (±9.01)	21.11 (±11.17)	0.284
Dihydrolanosterol (*μ*g/dL)	13.33 (±2.304)	14.65 (±4.385)	0.260
Desmosterol (mg/dL)	0.1401 (±0.052)	0.1295 (±0.067)	0.595
7*α*-OH-cholesterol (ng/mL)	72.33 (±101.0)	98.95 (±84.29)	0.382
Cholesterol (mg/dL)	185.4 (±27.51)	209.3 (±47.96)	0.071
27-OH cholesterol (ng/mL)	**185.0 (** ± **54.84)**	**135.0 (** ± **44.69)**	**0.004**
Cholestanol (mg/dL)	**0.2825 (** ± **0.060)**	**0.3501 (** ± **0.088)**	**0.010**
Brassicasterol (*μ*g/dL)	**32.07 (** ± **3.802)**	**11.16 (** ± **1.518)**	**<0.0001**

**Table 3:** Serum analytes in control and DS cohorts by age. Analytes shown in bold are significantly different between control and DS groups by unpaired student's *t*-test. Welch's correction was applied when variances were significantly different between groups.

	Con < 45	DS < 45	*P*	Con > 45	DS > 45	*P*
TC (mmol/L)	4.859 (±0.622)	5.095 (±1.050)	0.576	4.974 (±0.793)	4.811 (±1.201)	0.727
HDL-C (mmol/L)	1.632 (±0.632)	1.357 (±0.293)	0.340	1.643 (±0.410)	1.443 (±0.268)	0.204
ApoA-I (mg/mL)	1.447 (±0.298)	1.333 (±0.179)	0.362	**1.547 (** ± **0.196)**	**1.317 (** ± **0.188)**	**0.016**
ApoB (mg/mL)	0.826 (±0.196)	0.918 (±0.203)	0.358	0.780 (±0.162)	0.891 (±0.293)	0.320
CRP (mmol/L)	0.800 (±1.872)	2.743 (±2.045)	0.051	1.233 (±1.806)	2.938 (±2.286)	0.107
Campesterol (mg/dL)	0.5406 (±0.333)	0.4378 (±0.283)	0.547	**0.4894 (** ± **0.219)**	**0.3015 (** ± **0.114)**	**0.014**
Sitosterol (mg/dL)	0.437 (±0.238)	0.352 (±0.225)	0.506	** 0.422 (** ± **0.213)**	**0.2369 (** ± **0.102)**	**0.010**
24S-OH cholesterol	63.67 (±3.122)	66.50 (±15.20)	0.721	60.78 (±3.172)	71.36 (±7.038)	0.187
Lathosterol (mg/dL)	0.2039 (±0.115)	0.2703 (±0.092)	0.259	0.1951 (±0.085)	0.2054 (±0.141)	0.847
Campestanol (mg/dL)	3.639 (±1.704)	4.138 (±2.789)	0.672	3.659 (±0.990)	3.038 (±1.187)	0.207
Stigmasterol (mg/dL)	10.09 (±1.092)	13.99 (±3.103)	0.280	10.33 (±1.996)	11.04 (±3.360)	0.572
Sitostanol (mg/dL)	4.709 (±1.394)	5.338 (±3.018)	0.591	4.790 (±1.106)	3.921 (±1.183)	0.084
Lanosterol (mg/dL)	20.11 (±11.26)	21.80 (±10.60)	0.776	18.60 (±10.15)	20.81 (±11.79)	0.648
Dihydrolanosterol (mg/dL)	13.66 (±2.625)	14.25 (±4.562)	0.739	13.60 (±3.067)	14.81 (±4.473)	0.487
Desmosterol (mg/dL)	0.1522 (±0.063)	0.1413 (±0.058)	0.741	0.1352 (±0.047)	0.1244 (±0.073)	0.696
7*α*-OH-cholesterol (ng/mL)	86.89 (±47.13)	81.67 (±1.60)	0.919	57.78 (±34.59)	106.4 (±97.33)	0.167
Cholesterol (mg/dL)	**178.8 (** ± **22.16)**	**222.2 (** ± **32.25)**	**0.008**	192.3 (±30.57)	203.8 (±53.01)	0.564
27-OH cholesterol (ng/mL)	**219.1 (** ± **57.88)**	**135.0 (** ± **44.99)**	**0.010**	164.8 (±46.98)	135.0 (±46.27)	0.149
Cholestanol (mg/dL)	**0.2743 (** ± **0.070)**	**0.3727 (** ± **0.085)**	**0.022**	0.2976 (±0.059)	0.3404 (±0.091)	0.274
Brassicasterol (mg/dL)	**35.40 (** ± **17.83)**	**14.12 (** ± **10.90)**	**0.039**	**33.07 (** ± **19.02)**	**9.886 (** ± **3.99)**	**0.007**
